# The large universal *Pantoea* plasmid LPP-1 plays a major role in biological and ecological diversification

**DOI:** 10.1186/1471-2164-13-625

**Published:** 2012-11-15

**Authors:** Pieter De Maayer, Wai-Yin Chan, Jochen Blom, Stephanus N Venter, Brion Duffy, Theo H M Smits, Teresa A Coutinho

**Affiliations:** 1 Forestry and Agricultural Biotechnology Institute, Department of Microbiology and Plant Pathology, University of Pretoria, Pretoria, South Africa; 2 Center for Microbial Ecology and Genomics, Department of Genetics, University of Pretoria, Pretoria, South Africa; 3 CeBiTec, University of Bielefeld, Bielefeld, Germany; 4 Agroscope Changins-Wädenswil ACW, Division of Plant Protection, Wädenswil, Switzerland

## Abstract

**Background:**

*Pantoea* spp. are frequently isolated from a wide range of ecological niches and have various biological roles, as plant epi- or endophytes, biocontrol agents, plant-growth promoters or as pathogens of both plant and animal hosts. This suggests that members of this genus have undergone extensive genotypic diversification. One means by which this occurs among bacteria is through the acquisition and maintenance of plasmids. Here, we have analyzed and compared the sequences of a large plasmid common to all sequenced *Pantoea* spp.

**Results and discussion:**

The Large *Pantoea*Plasmids (LPP-1) of twenty strains encompassing seven different *Pantoea* species, including pathogens and endo-/epiphytes of a wide range of plant hosts as well as insect-associated strains, were compared. The LPP-1 plasmid sequences range in size from ~281 to 794 kb and carry between 238 and 750 protein coding sequences (CDS). A core set of 46 proteins, encompassing 2.2% of the total pan-plasmid (2,095 CDS), conserved among all LPP-1 plasmid sequences, includes those required for thiamine and pigment biosynthesis. Phylogenetic analysis reveals that these plasmids have arisen from an ancestral plasmid, which has undergone extensive diversification. Analysis of the proteins encoded on LPP-1 also showed that these plasmids contribute to a wide range of *Pantoea* phenotypes, including the transport and catabolism of various substrates, inorganic ion assimilation, resistance to antibiotics and heavy metals, colonization and persistence in the host and environment, pathogenesis and antibiosis.

**Conclusions:**

LPP-1 is universal to all *Pantoea* spp. whose genomes have been sequenced to date and is derived from an ancestral plasmid. LPP-1 encodes a large array of proteins that have played a major role in the adaptation of the different *Pantoea* spp. to their various ecological niches and their specialization as pathogens, biocontrol agents or benign saprophytes found in many diverse environments.

## Background

The enterobacterial genus *Pantoea* currently comprises nineteen species of Gram-negative, yellow or beige pigmented, motile rods [1]. Members of this genus have been isolated from a wide range of environments including soil, water, dust, dairy products, meat, fish, insects, humans and animals [2]. Most frequently they are found associated with a broad range of plant hosts, as non-pathogenic endophytes or epiphytes, colonizing the leaves, stems and roots [2,3]. In this context, some *Pantoea* strains can be beneficial to the plant host by contributing to growth promotion through processes such as the production of the plant-growth hormone indole-acetic acid (IAA), phosphate solubilization or nitrogen fixation [4,5]. Some *Pantoea* strains also provide effective protection to plants against various bacterioses as well as fungal diseases and postharvest fruit rots [6-8]. However, strains of several *Pantoea* species represent major plant pathogens themselves. *Pantoea stewartii* subsp. *stewartii* causes the devastating disease Stewart’s wilt of maize, and pathovars of *Pantoea agglomerans* which induce galls on gypsophila, beet, Douglas firs and wisteria [9,10]. *Pantoea* spp. are also frequently found associated with insects [11,12]. For example, the corn flea beetle *Chaetocnema pulicaria*, serves as the vector of the Stewart’s wilt pathogen *P. stewartii* subsp. *stewartii*[9,13]. Strains of some *Pantoea* species have been implicated as opportunistic human pathogens in cases of septicemia following penetrating trauma with plant material, nosocomial infections due to exposure to contaminated hospital materials and secondary pathogens complicating pre-existing illnesses [3,14,15].

The various ecological niches occupied by *Pantoea* species including both plant and animal hosts, and their distinctive lifestyles as epi- and endophytes, plant-growth promoters, biological control agents, or as pathogens of plant and animal hosts are indicative of extensive diversification within the genus *Pantoea* and even among individual strains belonging to the various *Pantoea* species. One means by which this diversification may be accomplished in bacteria is through the acquisition of plasmids, extra-chromosomal genetic elements capable of transfer between strains, species and genera and subsequent stable vertical transmission within the bacterial cell line [16]. These plasmids carry genes that can confer various phenotypes on the bacterium, including toxin, hormone production and virulence factors contributing to pathogenesis and host specificity, resistance to antibiotics and heavy metals and survival in adverse conditions, catabolism of amino and organic acids, carbohydrates and inorganic ions, colonization and dissemination [16,17]. Plasmid acquisition can thus contribute to both the survival of a bacterium in an existing environment and colonization of novel niches [17].

Plasmids are common features among *Pantoea* species. Strains of *P. stewartii* subsp. *stewartii* carry up to 13 plasmids which may contribute 25% of the total genome content [18]. *Pantoea* sp. At-9b, which is associated with leaf-cutter ants, carries five large plasmids which encompass approximately 31% of the total genome content [12]. The importance of these plasmids to the biology of *Pantoea* species is evident in *P. agglomerans* pvs. *betae* and *gypsophilae*, which carry a 150 kb pPATH plasmid which is absent from other, non-pathogenic, *P. agglomerans* strains. This plasmid encodes a Type III secretion system as well as enzymes for the synthesis of indole acetic acid which confer the ability on these pathovars to infect and induce tumorigenesis on various plant hosts [10]. The biological control agent *Pantoea vagans* C9-1, registered in the United States and Canada for control of the fire blight pathogen *Erwinia amylovora*, carries a plasmid, pPag2, which encodes for the biosynthesis of the antimicrobial peptide herbicolin I [8,19].

During early experiments with strains of several *Pantoea* species it was observed that extensive culturing and growth at supra-optimal temperatures result in the development of non-pigmented variants [20-22]. Biochemical analyses showed that these variants also lost the ability to synthesize the essential co-factor thiamine and could no longer utilize carbohydrate sources such as citrate and maltose [20,22,23]. The lack of reversion to a wild-type phenotype along with further basic molecular characterization indicated that these characteristics are encoded on a single large plasmid which has been lost in the non-pigmented variants [20,23]. With the availability of the complete genome sequence, Smits et al. [23] were able to characterize the 530-kb plasmid carrying these factors in *P. vagans* C9-1. This plasmid also carries genes for the biosynthesis of the siderophore desferrioxamine E, acyl-homoserine lactone biosynthesis and quorum sensing, ampicillin resistance and carbohydrate utilization pathways which may contribute to the ecological fitness and efficacy of this biocontrol strain [8,23].

The complete or draft genomes of 20 *Pantoea* strains belonging to seven distinct species have been sequenced to date. Here we elucidate and compare the sequences of the pigment biosynthesis and thiamine autotrophy-associated plasmid which we have given the collective name Large *Pantoea* Plasmid-1 (LPP-1). Our analyses reveal that LPP-1, which is common to these sequenced *Pantoea* strains, has been derived from an ancestral plasmid that has undergone extensive diversification. The variation in the LPP-1 plasmid sequences may be linked to various phenotypes that suggest this plasmid is an important driver of the biological, ecological and lifestyle diversification observed among the *Pantoea* species.

## Results and discussion

### Characteristics of the Large *Pantoea* Plasmid

**Figure 1:**
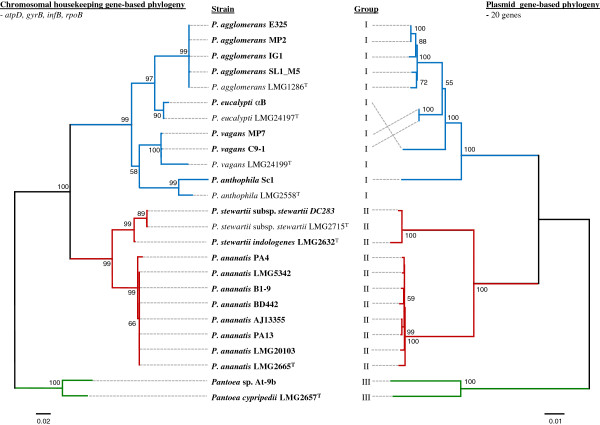
**Phylogeny of the *Pantoea *spp. based on chromosomal house-keeping genes and LPP-1 plasmid genes.** A neighbour-joining phylogeny constructed on the basis of the concatenated amino acid sequences of four chromosomal house-keeping genes, *atpD*, *infB*, *gyrB* and *rpoB* is shown on the left hand side. The type strains of those species for which no type strain genome sequences are available, were included. The neighbour-joining tree on the right hand represents the phylogeny constructed using the concatenated amino acid sequences for 20 proteins conserved on all Group I-III *Pantoea* spp. Bootstrap values are shown. The branches in both trees are coloured according to the groups, with Group I branches in blue, Group II in red and Group III in green

The LPP-1 plasmid sequences of 20 strains encompassing seven species within the genus *Pantoea* were analyzed (Table 1). These plasmids represent the largest extra-chromosomal elements in each of the sequenced organisms. The LPP-1 genomes range in size from 281 to 794 kb in size and thus contribute between 5.6% (*P. stewartii* subsp. *stewartii* DC283) and 12.6% (*Pantoea* sp. At-9b) of the total genome content. This indicates that LPP-1 contributes significantly to the *Pantoea* genotype. On the basis of a phylogeny using the amino acid sequences of four chromosomal house-keeping genes, the *Pantoea* strains used in this study could be separated into three distinct groups, Group I-III (Figure 1). Similarly, a pattern in terms of the LPP-1 size and G+C content (%) could be observed within each distinct group (Table 1). Group I encompasses members of the species *P. agglomerans, Pantoea eucalypti, P. vagans* and *Pantoea anthophila.* The Group I LPP-1 plasmid sequences have an average size 524-kb and G+C content of 53.90%. One notable exception is the *P. anthophila* Sc1 LPP-1 which is only 411 kb in size, with a G+C content of 56.27%. The LPP-1 of group II organisms *Pantoea ananatis* and *P. stewartii* has an average size of 310 kb with a G+C content of 51.75%. Group III incorporates the leaf-cutter ant-associated *Pantoea* sp. At-9b and the orchid pathogen *Pantoea cypripedii* LMG2657^T^ and their LPP-1 plasmid sequences have an average size of 720 kb and G+C content of 54.18%.

**Table 1:** **General characteristics of the LPP-1 plasmids of *Pantoea *strains included in the study**

**Group**	**#**	**Organism**	**Strain**	**Isolated from**	**Lifestyle**	**Sequence Status**	**Reference**	**Plasmid size (kb)**	**G+C (%)**	**# CDS**
I	1	*P. agglomerans*	E325	Apple	Biocontrol	Draft	[a]	535.8	53.52	552
I	2	*P. agglomerans*	IG1	Wheat	Saprophyte	Draft	[58]	616.4	52.65	578
I	3	*P. agglomerans*	MP2	Termite	Symbiont	Draft	[b]	560.6	53.18	527
I	4	*P. agglomerans*	SL1_M5	Woodwasp	Symbiont	Draft	[11]	526.2	53.53	499
I	5	*P. anthophila*	Sc1	Cotton	Pathogen	Complete	[59]	411.4	56.27	376
I	6	*P. eucalyptii*	αB	Bark beetle	Symbiont	Draft	[c]	499.6	54.2	492
I	7	*P. vagans*	C9-1	Apple	Biocontrol	Complete	[60]	529.7	53.87	474
I	8	*P. vagans*	MP7	Termite	Symbiont	Draft	[b]	508.6	53.98	453
II	1	*P. ananatis*	AJ13355	Soil	Saprophyte	Complete	[56]	321.7	51.97	272
II	2	*P. ananatis*	B1-9	Onion	Saprophyte	Draft	[57]	314.4	51.68	267
II	3	*P. ananatis*	BD442	Maize	Pathogen	Draft	[a]	352.8	51.13	320
II	4	*P. ananatis*	LMG20103	*Eucalyptus*	Pathogen	Complete	[54]	305.7	51.76	259
II	5	*P. ananatis*	LMG2665^T^	Pineapple	Pathogen	Draft	[a]	317.1	52.11	270
II	6	*P. ananatis*	LMG5342	Human	Pathogen	Complete	[55]	302.6	51.47	255
II	7	*P. ananatis*	PA4	Onion	Pathogen	Draft	[a]	312.7	52.17	263
II	8	*P. ananatis*	PA13	Rice	Pathogen	Draft	[53]	280.8	52.25	238
II	9	*P. stewartii* subsp. *indologenes*	LMG2632^T^	Foxtail millet	Pathogen	Draft	[a]	301.9	51.44	265
II	10	*P. stewartii* subsp. *stewartii*	DC283	Maize	Pathogen	Draft	[c]	294.6	51.51	267
III	1	*Pantoea* sp.	At-9b	Leaf cutter ant	Symbiont	Complete	[c]	793.9	54.58	750
III	2	*P. cypripedii*	LMG2657^T^	Orchid	Pathogen	Draft	[a]	645.5	53.78	571

The host or site of isolation, the lifestyle as symbiont, saprophyte, biocontrol agent or pathogen and the status of the genome sequence for each of the 20 *Pantoea* strain included in this study, as well as the LPP-1 group they belong to are shown. Characteristics of the LPP-1 plasmids, including the genome size (in kilobases), G+C content (%) and number of protein coding sequences (CDS) encoded on each LPP-1 plasmid are given. In the Reference column [a] denotes those genome sequences which were obtained in our laboratories, while [b] denotes those provided by Poulsen et al., University of Copenhagen, Denmark. References labelled [c] are those draft or complete genomes that are publically available in the NCBI database, which have not been published. These include *Pantoea* sp. At-9b (NC_014838) and *Pantoea eucalypti* αB (AEDL00000000) submitted by Lucas et al. and *P. stewartii* subsp. *stewartii* DC283 (AHIE00000000) submitted by Biehl et al.

### The large *Pantoea* plasmids are derived from a common ancestor

**Figure 2:**
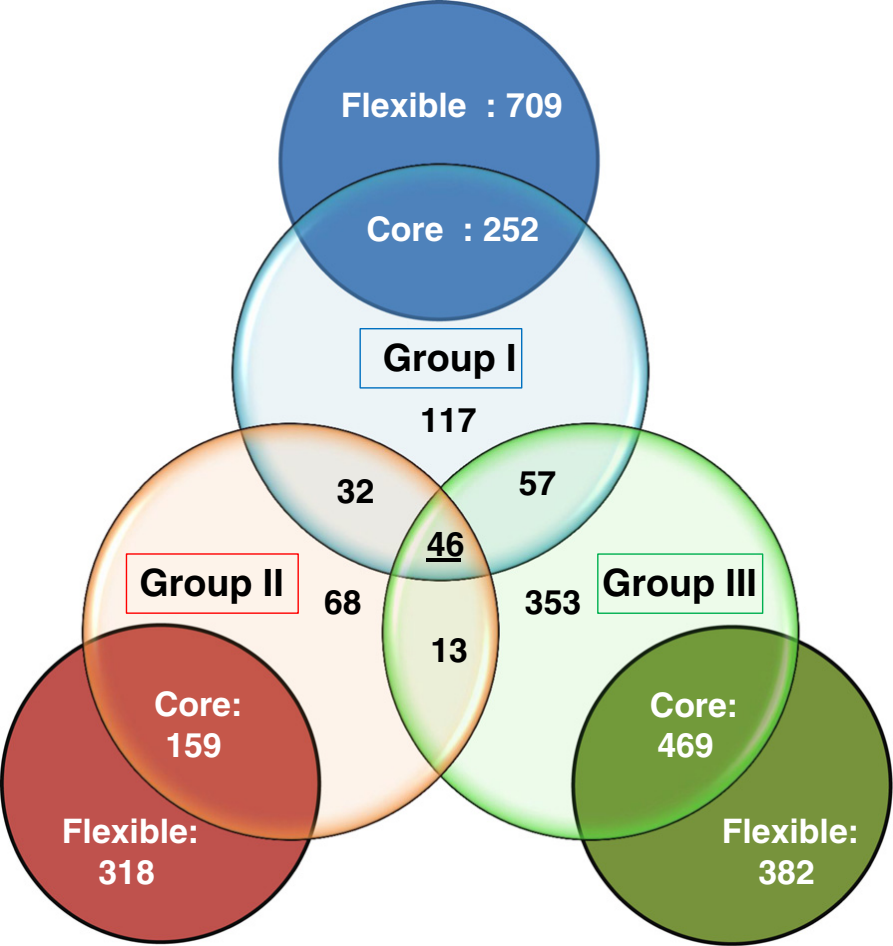
**Venn diagram of the *Pantoea *spp. LPP-1 pan-genome.** The outer circles represent the LPP-1 pan-genomes for Group I (blue), Group II (red) and Group III (green) and show the conserved (core) and non-conserved (flexible) CDS for each group. The core CDS were further compared as shown in the inner Venn diagram, with underlined value in the centre representing the conserved core CDS for all LPP-1 genomes

The LPP-1 CDS sets for each organism within each separate group were compared by BlastP analyses. By this means, the LPP-1 pan-genome for each group, as well as the conserved core and flexible portions were determined (Figure 2). The core CDS sets for each group were then further compared in order to determine those CDS common to all LPP-1 plasmids. A total of 46 CDS are conserved among the LPP-1 plasmids of all 20 strains (Additional file 1: Table S1, Figure 2). Included among these are five genes, *crtEYIBZ*, whose translation products are involved in the production of the yellow carotenoid pigment zeaxanthin [24]. This secondary metabolite has been shown to protect the producing organism against UV irradiation and phototoxic damage [25,26]. An additional gene, *crtX*, whose product adds a diglucoside group to the pigment is absent from the LPP-1 plasmids of Group III. Also common on all LPP-1 plasmids are the *thiOSGF* genes required for biosynthesis of the essential co-factor thiamine [27]. *Pantoea* strains lacking this plasmid have been demonstrated to require thiamine supplementation for growth [23]. Other conserved genes include those encoding AscBFG involved in the transport and catabolism of arbutin, salicin and cellobiose, the Dat and Ddc enzymes intermediate in the the biosynthesis of 1,3-diaminopropane and the enzyme malate:quinone oxidoreductase (Mqo) involved in the tricarboxylic acid cycle.

A phylogeny based on the concatenated amino acid sequences of 20 LPP-1 core CDS was constructed (Figure 1). This phylogeny shows a good correlation with that of the concatenated amino acid sequences of the four chromosomal house-keeping markers *atpD*, *gyrB*, *infB* and *rpoB* in terms of the clear taxon delineation into Groups I-III observed in both phylogenies (Figure 1). Furthermore, the taxon separation within each group in the LPP-1 tree largely matches those based on house-keeping genes present on the chromosome. This indicates that the LPP-1 plasmid may have been derived from an ancestral plasmid which shares a similar evolutionary history as the chromosomes of the associated *Pantoea* spp. As it has been observed that LPP-1 carries genes necessary for the production of the essential co-factor thiamine, and the selective advantage which would be provided by the carotenoid pigment in organisms which are frequently associated with UV-exposed surfaces of plants, strengthens the argument for vertical inheritance.

### LPP-1 has undergone extensive diversification among the *Pantoea* spp

**Figure 3:**
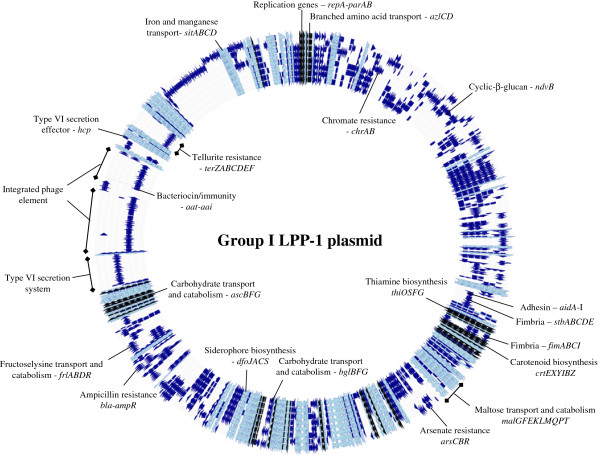
**Genome diagram of the Group I LPP-1 pan-genome.** Diagram showing the major phenotypes encoded on the Group I LPP-1 pan-genome. The circles represent the LPP-1 genomes of the *Pantoea* strains (from inside to outside): 1. *P. agglomerans* E325, 2. *P. agglomerans* MP2, 3. *P. agglomerans* IG1, 4. *P. agglomerans* SL1_M5, 5. *P. vagans* MP7, 6. *P. vagans* C9-1, 7. *P. eucalypti* αB and 8. *P. anthophila* Sc1*.* CDS in the flexible portion of Group I pan-genome are colored dark blue, while those in conserved among all Group I LPP-1 genomes are colored light blue. Black arrows indicate those CDS conserved among all 20 LPP-1 genomes

**Figure 4:**
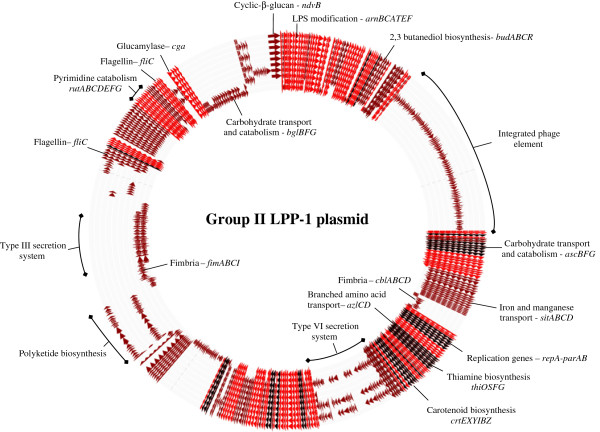
**Genome diagram of the Group II LPP-1 pan-genome.** Diagram showing the major phenotypes encoded on the Group II LPP-1 pan-genome. The circles represent the LPP-1 genomes of the *Pantoea* strains (from inside to outside): 1. *P. stewartii* subsp. *stewartii* DC283, 2. *P. stewartii* subsp. *indologenes* LMG2632^T^, 3. *P. ananatis* PA4, 4. *P. ananatis* LMG5342, 5. *P. ananatis* B1-9, 6. *P. ananatis* BD442, 7. *P. ananatis* AJ13355, 8. *P. ananatis* PA13, 9. *P. ananatis* LMG20103 and 10. *P. ananatis* LMG2665^T^. CDS in the flexible portion of Group II pan-genome are colored maroon, while those in conserved among all Group II LPP-1 genomes are colored red. Black arrows indicate those CDS conserved among all 20 LPP-1 genomes

**Figure 5:**
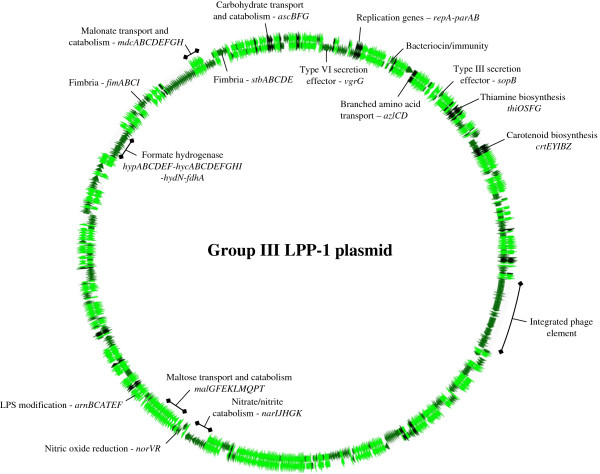
**Genome diagram of the Group III LPP-1 pan-genome.** This diagram shows the major phenotypes encoded on the Group III LPP-1 pan-genome. The inner circular track represents *Pantoea* sp. At-9b, while the outer track represents the LPP-1 genome of *P. cypripedii* LMG2657^T^. CDS in the flexible portion of Group III pan-genome are colored dark green, while those in conserved among all Group III LPP-1 genomes are colored light green. Black arrows indicate those CDS conserved among all 20 LPP-1 genomes

While there are 46 CDS conserved among the LPP-1 of all 20 *Pantoea* strains, this only represents a small fraction (2.2%) of the total pan-plasmid (2,095 CDS) (Figure 2). This indicates that although they are derived from a common ancestral plasmid, the LPP-1 plasmids of the different *Pantoea* strains have undergone massive genetic diversification. Even within the different groups, the extensive plasticity of the LPP-1 plasmid sequence can be observed. Among *Pantoea* Group I strains, the flexible portion of the pan-plasmid (709 CDS) (73.8%) makes up 73.8% of the pan-plasmid (Figures 2 and 3), while in Group II, this portion contributes 66.7% (318 CDS) of the pan-plasmid CDS (Figures 2 and 4). In Group III, where only two closely related strains were compared, the flexible CDS encompasses 44.8% (382 CDS) of the total (Figures 2 and 5). The extensive mosaicism of the Group I-III pan-plasmids can also be observed in Figures 3,5, with multiple non-conserved genomic regions inserted into the LPP-1 backbone. Many of these non-conserved regions show evidence that they represent mobile genetic elements, such as transposable and insertion elements. Furthermore, phage elements are integrated into the Group I, II and III LPP-1 plasmids of *P. agglomerans* IG1, *P. ananatis* BD442 and *Pantoea* sp. At-9b (Figures 3,5). There is also evidence of the exchange of genetic elements between the chromosomes and the LPP-1 plasmids of the *Pantoea* strains of the different groups. The maltose locus, *malGFEKLMQPT*, located on the LPP-1 of all Group I and III strains is maintained on the chromosome of Group II strains. Type VI secretion systems showing high sequence identity to those encoded on the LPP-1 plasmid of three Group II strains and *P. vagans* MP7 (Figures 3 and 4) have also been observed on the chromosomes of *P. eucalypti* αB, *P. agglomerans* E325 and *Pantoea* sp. At-9b [28]. There is also evidence for chromosome/LPP-1 exchange within the groups. For example, the *pagRI* genes, required for the production of the acyl-homoserine lactone autoinducer PagI and the transcriptional regulator PagR involved in the regulation of ecological fitness and virulence genes in various *Pantoea* spp. and related taxa, are chromosomally localized in most of the Group I strains, but are located on the plasmid of *P. vagans* strains C9-1 and MP7 [23].

### LPP-1 plays a major role in the diversification of *Pantoea* spp

The functions of the core and flexible CDS encoded on the various LPP-1 plasmids were determined on the basis of sequence similarity to characterized proteins. This indicated that the LPP-1 plasmids carry genes which may influence a variety of phenotypes. These phenotypes, which are discussed in further detail below, include the ability to take up and utilize a wide range of carbohydrate, amino acid and organic acid substrates as well as inorganic ions, resistance to antibiotics and heavy metals, as well as encoding potential pathogenicity and antibiosis factors. The LPP-1 plasmid may thus contribute extensively to the ability of *Pantoea* spp. to colonize the various ecological niches and hosts from which they have been isolated, as well as their different lifestyles as plant endo- or epiphytes, biocontrol agents and pathogens (Additional file 1: Table S1).

#### LPP-1 contributes to Pantoea metabolic versatility

White, plasmid-cured variants of *P. vagans* C9-1 have been shown to be unable to efficiently utilize a number of carbohydrate, amino and organic acid substrates, thus indicating a role for the plasmid in the metabolic versatility of this organism [23]. Analysis of the LPP-1 plasmid sequences of the 20 strains showed that this may represent a general trend for the plasmid, as a large number of loci for the transport and utilization of these metabolic substrates are encoded on LPP-1 (Additional file 1: Table S1). Common to all LPP-1 plasmids is a locus, *ascBFG*, which plays a role in the transport and catabolism of the sugars arbutin, salicin and cellobiose [29]. Other transport and metabolism-associated loci are more restricted in their distribution among the LPP-1 plasmids. An additional locus for the transport of these sugars, *bglBFG*, is found in most Group I and III strains, but not in strains of *P. ananatis*, *P. eucalypti* αB or *P. vagans* MP7. A maltose/maltodextrin transport and catabolism locus is found in all Group I and III organisms [23], but not those of Group II, where a locus with high sequence identity is located on the chromosome. Other carbohydrate transport and metabolism-associated loci encoded on the LPP-1 plasmid sequence, include those for galactan (*ganKEFGABCLR*), a major constituent of the pectic cell wall polymers found in plant cell walls [30], found in all Group I strains except *P. anthophila* Sc1, malonate (*mdcABCDEFGH*) encoded on Group III LPP-1 plasmids and a locus (*frlABDR*) for the catabolism of the glucose/amine agglomerates fructoselysine and psicoselysine [31] encoded on the *P. agglomerans* MP2 LPP-1. The LPP-1 of all *Pantoea* Group II strains also encodes a glucoamylase (Gca), which can be used to release glucose from starch, a major energy storage compound in plants [32].

A number of loci for the transport and catabolism of amino acid and organic acid substrates are also encoded on the Group I-III LPP-1 plasmids, including those for the transport of branched amino acids (*azlCD*) found in Group I-III, tyrosine (*tutB-tpl*) in *P. ananatis* strains and a tryptophan-specific transporter (*mtr*) encoded on the LPP-1 plasmids of *P. vagans* MP7 and *P. eucalypti* αB. A D-methionine transporter is also encoded on the LPP-1 of most *Pantoea* Group I strains. While these represent loci which show extensive homology to characterized transport and catabolism systems for known substrates, the uptake and utilization of a large number of other uncharacterized substrates may be linked to the LPP-1 plasmids. For example, a further 41 putative transport systems for unknown substrates were identified in the LPP-1 plasmid of *Pantoea* sp. At-9b. This further supports the important role of LPP-1 in the metabolic versatility of *Pantoea* spp.

#### LPP-1 plays a role in iron and nitrogen assimilation

The LPP-1 plasmid sequences also encode several proteins involved in the assimilation of the essential elements iron and nitrogen, which are commonly found in limited supply in the environment. In order to scavenge iron from the environment or from their host, bacteria use high-affinity iron-binding molecules known as siderophores [8,33]. A desferrioxamine E siderophore biosynthetic locus, *dfoJACS* is encoded on all Group I LPP-1 plasmids, while this locus is found on the chromosome of *Pantoea* Group II strains [33]. Iron-siderophore complexes are subsequently captured at the bacterial cell surface by high-affinity TonB-dependent receptor proteins, and translocated into the periplasm by the Ton system before being transported into the cytoplasm via inner membrane-associated ABC transporters [34]. Nine outer membrane receptor proteins without cognate siderophore biosynthetic systems are encoded on the LPP-1 plasmids (Additional file 1: Table S1). These can be utilized to scavenge iron-loaded siderophores produced by other microorganisms occupying the same ecological niches. This xenosiderophore scavenging will thus allow the acquisition of siderophore-bound iron without the energy cost of siderophore production [35]. An inner membrane iron and manganese transporter, SitABCD is also encoded on the LPP-1 of all Group I and II strains, with the exception of *P. stewartii* subsp. *stewartii* DC283.

The Rut pathway has been shown to release nitrogen through the catabolism of pyrimidine nucleotides [36]. The *rutRABCDEFG* locus encoded on the LPP-1 of all Group II strains may thus aid in the assimilation of nitrogen. A locus, *narIJHGK*, in the Group III strain *P. cypripedii* LMG2657^T^ can reduce nitrate to nitrite which can act as terminal respiratory electron acceptor [37]. An ortholog of NorV, a flavorubredoxin involved in the detoxification of the nitric oxide, a cytotoxic byproduct of nitrate reduction [37] is also encoded on the LPP-1 of this organism.

#### LPP-1 contributes to antibiotic and heavy metal resistance

A β-lactamase *bla* and its cognate transcriptional regulator *ampR* have been identified on the LPP-1 plasmid of *P. vagans* C9-1, and their translation products were shown to be required for ampicillin resistance [8,23]. Genes showing extensive sequence identity to *bla-ampR* are present on the LPP-1 plasmids of the Group I strains *P. agglomerans* IG1, *P. anthophila* Sc1 and *P. vagans* MP7. The LPP-1 of *P. vagans* MP7 also carries a locus *terZABCDEF*, which is localized on the pPag1 plasmid of *P. vagans* C9-1 [19]. This locus is required for resistance to tellurite, a rare oxide mineral which has a long history as antimicrobial agent [38]. Six distinct RND multidrug efflux pumps are also encoded on the Group I-III LPP-1 plasmids (Additional file 1: Table S1). Furthermore, all Group II and III LPP-1 plasmids carry a locus (*arnBCATEF*) and a further protein PagO encoded on the LPP-1 of Group III *Pantoea* spp., which are involved in the addition of 4-amino-4-deoxy-L-arabinose and palmitate side chains to the lipopolysaccharide lipid A moiety, respectively. These modifications confer resistance to polymyxin and cationic antimicrobial peptides [39,40]. Thus LPP-1 encodes various proteins which may play a role in protecting the different *Pantoea* species against various antimicrobials. LPP-1 also carries loci which can confer resistance to heavy metals. Loci showing extensive sequence conservation to those involved in resistance to arsenate (*arsCBR*) are located on the LPP-1 plasmids of four Group I *Pantoea* strains, while a chromate resistance locus (*chrAB*) is carried on the *P. vagans* MP7 LPP-1.

#### LPP-1 may play a role in host colonization and persistence

A total of seven distinct fimbriae are encoded among the twenty LPP-1 plasmids which were compared, with fimbrial loci present on the LPP-1 plasmids of representatives of all three groups (Additional file 1: Table S1). *P. stewartii* subsp. *indologenes* LMG2632^T^ LPP-1 encodes a *cblABCD* locus required for the synthesis of cable pili, which have been shown to play an important role in the attachment of the cystic fibrosis pathogen *Burkholderia cepacia* to epithelial cells and persistence in the airways [41]. The LPP-1 plasmid of *P. agglomerans* IG1 also carries a gene encoding a putative AidA-like autotransporter adhesin. The presence of genes for these adhesive structures on LPP-1, suggests this plasmid plays a role in the attachment and persistence of *Pantoea* in the different environmental niches they occupy, including potentially the various plant, insect and vertebrate hosts from which they have been isolated.

The Group II LPP-1 also carries two *fliC* genes encoding flagellin, the major subunit of flagella. These *fliC* genes are not located adjacent to the remainder of the *fli* locus required for flagellum biosynthesis, which is present on the chromosome next to a further *fliC* copy. Multiple *fliC* copies have been found encoded on the plasmids of a number of bacteria, including *Salmonella enterica* and *Escherichia coli*[42,43]. As the flagellin protein represents one of the major surface antigens in Gram-negative bacteria which when recognized by the host triggers defense responses, the alternate expression of different copies of *fliC*, termed phase variation, can enable the bacterium to evade host defenses [42,43]. The LPP-1 plasmid sequences of the Group II *P. ananatis* strains as well as *P. vagans* C9-1 encode a protein NdvB required for the synthesis the extracellular cyclic β-glucan polymer [44]. In the phytopathogen *Xanthomonas campestris* pv. *campestris*, this glucan has been shown to be spread throughout the host plant and suppress both systemic and localized host defenses [44]. By contrast, with the exception of *P. stewartii* subsp. *stewartii* DC283, all Group II *Pantoea* strains carry a locus, *budABCR*, involved in the biosynthesis of the bacterial volatile 2,3-butanediol, which has been demonstrated to trigger systemic resistance in plants [45,46]. This may correlate with the role as plant-growth promoter which has been identified for some *Pantoea* spp. [4,5].

The LPP-1 plasmid sequence of *Pantoea* sp. At-9b also carries an extensive locus for the synthesis and maturation of a formate hydrogenase (Additional file 1: Table S1, Figure 5). This enzyme enables a bacterium to exploit hydrogen as an energy source under anaerobic conditions and has been found to play a major role in gut colonization and virulence in enteric pathogens [47]. As *Pantoea* sp. At-9b has been found in close association with leaf-cutter ants [12], this hydrogenase may play a role in its colonization of an insect host.

#### LPP-1 encodes putative pathogenicity and antibiosis factors

The Hrp Type III secretion system represents one of the main pathogenicity determinants of the corn pathogen *P. stewartii* subsp. *stewartii*, playing a role in both systemic infection and the development of the water-soaked lesions typical of Stewart’s wilt of corn [9,13]. The LPP-1 plasmid sequences of the Group II strains *P. stewartii* subsp. *stewartii* DC283 and *P. stewartii* subsp. *indologenes* LMG2632^T^ incorporate a Hrp locus. The latter organism is thought to cause leaf spot of pearl and foxtail millet and rot of pineapple [48]. The presence of the Hrp T3SS on the *P. stewartii* LPP-1 suggests this plasmid is thus required for pathogenesis on their different plant hosts.

Encoded on the LPP-1 of three *P. ananatis* strains (Additional file 1: Table S1 and Figure 4) as well as termite symbiont *P. vagans* MP7 is a Type VI secretion system (T6SS). Early studies indicated that this secretion system plays a major role in virulence in both animal and plant pathogens [28,49]. However, the presence in many potentially non-pathogenic bacteria and inconsistencies in experimental data have led to a number of different functions being postulated for this secretion system. One function recently identified for the T6SS of a number of different bacterial taxa is the secretion of antimicrobial effectors, which suggests it may play a role in antibiosis and inter-bacterial competition [49]. The function of the T6SS in the various *Pantoea* spp. still needs to be elucidated.

Located on the LPP-1 of *P. agglomerans* IG1 and *Pantoea* sp. At-9b are genes encoding a predicted bacteriocin and its respective immunity protein showing highest sequence identity to Alveicin A (*aat-aai*) produced by *Hafnia alvei* and Klebicin B (*kba-kbi*) produced by *Klebsiella pneumoniae*, respectively [50,51]. These bacteriocins are small toxic proteins, frequently encoded on plasmids, which can kill closely related bacteria [51]. The LPP-1 plasmids of the Group II strains *P. ananatis* B1-9 and LMG2665^T^ also carry a ~25.5-kb locus encoding proteins showing low sequence similarity to polyketide synthases (PKS)/non-ribosomal peptide synthases (NRPS). These proteins are involved in the synthesis of a wide range of structural variable secondary metabolites including antibiotics (e.g. vancomycin and penicillin), siderophores (e.g. yersiniabactin and pyoverdine) as well as toxins (e.g. the *Pseudomonas syringae* phytotoxin syringomycin) [52]. However, due to the low sequence similarity to characterized PKS/NRPS proteins, further characterization of the compounds produced by this locus in the *P. ananatis* strains will need to be performed before a definitive function can be assigned.

## Conclusions

The acquisition and maintenance of plasmids by bacteria has been demonstrated to play a key role in their adaptation to novel ecological niches and their development as symbiont, plant-growth promoters, saprophytes, biocontrol agents or pathogens [16]. Our analysis of the Large *Pantoea* plasmid LPP-1 common to all members of the genus *Pantoea* sequenced to date, showed that similarly, this plasmid is likely to have played a major role in the ecological and biological adaptation of *Pantoea* spp. A core set of genes conserved on all LPP-1 plasmids include those required for thiamine biosynthesis, an essential cofactor for bacterial growth and survival. The maintenance of these essential genes on LPP-1, correlate with our phylogenetic analysis, suggesting that this plasmid must be stably inherited and maintained by *Pantoea* spp. and has thus evolved from a common ancestral plasmid. The backbone of LPP-1 has however been permeated, through the incorporation of a multitude of genetic elements to give rise to the extremely variable LPP-1 plasmids which can be observed for the various *Pantoea* spp. These variable elements include genes for the transport and catabolism of various metabolic substrates, the assimilation of inorganic ions, and resistance to antimicrobial peptides and heavy metals. These factors may in turn have provided the *Pantoea* spp. with the selective advantage to thrive in the vast array of ecological niches they occupy. Furthermore, the LPP-1 plasmid sequences encode proteins for the synthesis of several putative plant colonization, plant-growth promoting, antimicrobial and pathogenicity factors, which may have enabled their specialization as epi- and endophytes, effective biocontrol agents, as insect endosymbionts and as pathogens of plant and animal hosts. The LPP-1 plasmid thus represents a major evolutionary driver among the ecologically and biologically robust *Pantoea* spp.

## Methods

### Assembly and annotation of LPP-1

The genome sequences of 20 *Pantoea* spp. isolated from different environmental sources and hosts were used in this study (Table 1). These included nine published complete and high-quality draft genomes and eleven unpublished draft genomes sequenced, assembled and annotated in our laboratories or which are publically available [11,12,53-60]. The LPP-1 nucleotide sequences for the complete *Pantoea* genomes were used to assemble the LPP-1 plasmid for those organisms for which draft genomes are available. Draft contigs were aligned against the complete LPP-1 sequences of the nearest phylogenetic relatives using progressiveMauve [61]. Co-linear contigs were assembled to high quality draft LPP-1 sequences. Local BlastN analyses with the BioEdit v 7.0.5.3 software package [62] were performed using the thiamine biosynthesis locus *thiOSFG* and the carotenoid biosynthetic locus *crtEXYBIZ* to confirm that the plasmids belonged to the LPP-1 group. The CDS for complete LPP-1 sequences were downloaded from the National Center for Biotechnology Information (NCBI) database [63] and those encoded on the draft plasmids were predicted using FgenesB [64]. The CDS sets for each LPP-1 plasmid were standardized by performing reciprocal local BlastN analyses using BioEdit [62] to identify and add CDS which were not predicted in a particular LPP-1 plasmid sequence. BlastP analysis [65] against the NCBI protein database [63], as well as annotation of the LPP-1 CDS using the GenDB pipeline [66], were used to predict their function on the basis of sequence similarity to characterized proteins. Transport-associated proteins were further characterized by comparison against the Transporter Classification Database (TCDB) [67].

### Comparative analyses of the LPP-1 plasmids

The LPP-1 plasmids were categorized into three groups consistent with the phylogenetic clusters identified for the producing *Pantoea* strains on the basis of four chromosomal loci as described below. The LPP-1 pan-genomes for each of these groups were determined. This was done by local BlastP analysis of the LPP-1 CDS sets using a reciprocal best hit (RBH) approach [68]. Homologs were considered when CDS shared more than 70% amino acid identity over 70% of the average length of the two proteins. Comparative diagrams for each group incorporating the pan-genomes and CDS sets for all LPP-1 plasmids were constructed using GenomeDiagram [69]. The core CDS set, those that are common to all LPP-1 plasmids in each group, as well as the flexible CDS set were determined. The core CDS sets for each group were further compared using the RBH parameters described above. By this means, the CDS set which is core to all LPP-1 plasmids was determined.

### Phylogenetic analyses

A phylogeny based on the amino acid sequences for four chromosomal house-keeping genes, *atpD*, *gyrB*, *infB* and *rpoB* was constructed. Similarly, the amino acid sequences of 20 distinct plasmid-borne genes which are found in the overall LPP-1 core were used to determine the phylogenetic relationships of the large *Pantoea* plasmids. These included the thiamine biosynthetic locus *thiOSGF*, pigment biosynthesis locus *crtEYIBZ*, carbohydrate transport and utilization locus *ascBFG*, branched amino acid transporter *azlCD*, malate:quinone oxidoreductase *mqo*, 1,3-diaminopropane biosynthetic genes *dat-ddc* and the replication genes *repA* and *parAB*. A ClustalW alignment for the concatenated amino acid sequences was performed using the parameters as previously described [28]. Neighbour-joining phylogenetic trees (Poisson correction; complete gap deletion; bootstrap analysis – n = 1,000) were constructed using the Molecular Evolution Genetics Analysis (MEGA) v 5.0.3 software package [70].

## Competing interests

The authors declare that they have no competing interests.

## Authors’ contribution

PDM, SNV, BD, THMS and TAC conceived the study. PDM, WYC, JB and THMS performed experiments and analysis, PDM, SNV, BD, THMS and TAC wrote the original manuscript. All authors contributed to the final version.

## Supplementary Material

Additional file 1 **Table S1.** Phenotypes encoded on the LPP-1 genomes of Group I-III *Pantoea* spp. Table showing the phenotypes, as well as the genetic loci for these phenotypes, encoded on the LPP-1 genomes of Group I, II and III *Pantoea* strains. The presence/absence of a particularly genetic locus and phenotype in each Group is indicated by a + or -, respectively, and the strain number designations follow those indicated in Table 1. The phenotypes are further subdivided into groups according to their function, including those factors which are conserved (core) among all twenty *Pantoea* spp., and those potentially involved in transport and catabolism of metabolic substrates, inorganic ion assimilation, resistance to antimicrobials and heavy metal, host colonization and persistence and putative pathogenesis and antibiosis factors. Click here for file
